# Prevalence of *Schistosoma mansoni*, soil-transmitted helminths and intestinal protozoa in orphans and street children in Mwanza city, Northern Tanzania

**DOI:** 10.1007/s15010-023-01999-9

**Published:** 2023-02-20

**Authors:** Anemone Franz, Antje Fuss, Humphrey D. Mazigo, Deodatus Ruganuza, Andreas Müller

**Affiliations:** 1 Julius-Maximilians-Universität Würzburg, Medicine, Würzburg, Germany; 2 Medical Mission Institute, Biology, Würzburg, Germany; 3 Medical Parasitology and Entomology, Catholic University of Health and Allied Sciences, Mwanza, United Republic of Tanzania; 4 Tropical Medicine, Klinikum Würzburg Mitte, Würzburg, Germany

**Keywords:** Mass drug administration, Parasitic infections, Schistosomiasis, Street children, Orphans

## Abstract

**Background:**

Parasitic infections are highly prevalent in low-income environments worldwide. While orphans and street children represent a particularly vulnerable population group, they are often exempt from preventive interventions such as Mass Drug Administration. In part, this could be due to a lack of data showing the burden of disease in this group. This study aims to address this gap.

**Methods:**

For this cross-sectional study, 144 orphans and 112 street children were screened for *Schistosoma mansoni* (*S. mansoni*), *Schistosoma haematobium* (*S. haematobium*), soil-transmitted helminths and intestinal protozoa using POC-CCA testing, urine filtration, and Kato-Katz technique. Nutritional status, water- and washing patterns were determined using a standardised questionnaire. Ultrasonography was performed to screen for organ abnormalities.

**Results:**

The prevalence of *S. mansoni* determined by POC-CCA-test was 65.9% for orphans and 94.5% for street children. 19.2% of the orphans tested positive for *S. mansoni* in Kato Katz. Of the street children, 77.1% showed positive test results in Kato-Katz. Only 1.3% of the orphans stated in the questionnaire that they use the lake to wash, whereas 91.1% of the street children named the lake as at least one of their options for washing. Microscopy showed positive results for *Giardia intestinalis* (*G. intestinalis*) in 8.2% and for *Entamoeba histolytica/dispar* (*E. histolytica/dispar*) in 23% of orphans and 8.1% for *G. intestinalis,* and 23.8% for *E. histolytica/dispar* in street children. In the ultrasonography, we did not observe patterns that indicate severe periportal fibrosis.

**Conclusion:**

The results indicate a significantly higher rate of infections with *S. mansoni* in street children compared with orphans. This might be explained by the lack of access to adequate sanitation for street children as well as regular contact with the water of Lake Victoria. However, we did not find similar results concerning infection rates with protozoa. The study results show overall inadequate living conditions in this study population, which could be addressed by public health interventions.

## Background

Parasitic worm infections are responsible for high morbidity and mortality worldwide and predominantly affect people in low-resource settings.

Schistosomiasis, which affects over 200 million people worldwide, is one of the neglected tropical diseases (NTD) with the highest impact on the global burden of disease [1]. In sub-Saharan Africa, Schistosomiasis is caused by *Schistosoma mansoni* (*S. mansoni*) and *Schistosoma haematobium (S. haematobium)*, which lead to intestinal and urogenital Schistosomiasis respectively. Chronic infections with *S. mansoni* can cause periportal fibrosis, hepatosplenomegaly, hematemesis and ascites [2–4]. Infections with *S. haematobium* can cause cystitis and urethritis, which can eventually lead to kidney failure and bladder cancer [3, 4]. In children, infections can cause anaemia, malnutrition, and stunting, which can impair their general development [5]. It has been shown that chronic infections reduce cognitive functions in children and can thus lead to decreased performance at school and later in the workplace [5–7]. Tanzania is a country with an exceptionally high prevalence of Schistosomiasis with infection rates of over 90% of school-aged children (SAC) on the shores of Lake Victoria [8].

Soil-transmitted helminths (STH), including *Ascaris lumbricoides (A.lumbricoides)* and *Trichuris trichiura* (*T. trichiura*) are nematode worms that infect humans through contact with parasite larvae and eggs. Infections with *A. lumbricoides* can cause a variety of symptoms like eosinophilic pneumonia, acute pancreatitis and weight loss [9]. Most common symptoms of infections with *T. trichiura* are abdominal pain, asthenia, diarrhoea and in severe cases trichuris dysentery syndrome [9]. In 2018, the World Health Organization (WHO) estimated that over 300 million pre-school-aged children required preventive chemotherapy for STH in 94 countries or territories [10].

To estimate hygiene conditions, infection rates with intestinal protozoa can be used. A high prevalence of *Giardia intestinalis (G. intestinalis)* and *Entamoeba histolytica/dispar (E. histolytica/dispar)* are strongly associated with inadequate water, sanitation and hygiene behaviour [11].

To treat parasitic infections and interrupt infection cycles, mass drug administration can be used. Treatments with Albendazole and Praziquantel are safe, effective and economical but must be repeated regularly, otherwise infection rates return to pre-treatment levels within a few months [12–14]. The WHO recommends treating all SAC in areas with a prevalence ≥ 50% of Schistosomes and STH annually, respectively, biannually [12]. Mass drug administration campaigns often use the existing infrastructure of schools. This ensures that individuals at a critical stage of physical and cognitive development can be reached and helps to keep this intervention cost-effective. However, some groups of children that do not regularly attend school will not be reached through these measures. This includes orphans, street children or children who work to support their families.

There is substantial research showing the prevalence of parasitic infections worldwide. However, the literature mostly excludes vulnerable subpopulations, like orphans and street children. This study aims to generate a better understanding of their morbidity and living conditions to streamline future targeted intervention programs.

## Methods

### Study site

Mwanza city was chosen as study site due to the high prevalence of helminths and protozoa in the general population. Tanzania is the country with the second highest prevalence of Schistosomiasis worldwide [15]. The city is located on the shorelines of Lake Victoria where especially high infection rates with Schistosomes have been observed. The lake is a natural habitat for the intermediate hosts for Schistosomes, namely freshwater snails *Biomphalaria choanomphala* and *Biomphalaria sudanica,* hosts for *S. mansoni,* and, to a lesser extent, *Bulinus species*, hosts to *S. haematobium* [16, 17]. For many people in this region, Lake Victoria is the primary source of fresh water.

Mwanza city is home to a very high number of orphans and street children. Railway children, an UK-based NGO, identified 1548 children living and working on the streets of Mwanza city during daytime and 738 during night-time in their headcount 2014 [18].

### Study design, sample size, inclusion and exclusion criteria

The cross-sectional study was conducted in March 2019. We included 144 orphans from four different orphanages (SOS, Fonelisco, Village of Hope, Wote Sawa). 70 children were male (48.6%), 68 female (47.2%) and 6 unknown (4.2%) In addition, 112 street children (97.3% male, 2.7% unknown) were screened. Contact with street children was established with the help of the NGO “Tanzania Rural Health Movement”. On three different days, children could freely present themselves to be included in the study.

Study participants were included if they

(i) Were permanent inhabitants of Mwanza city.
(ii) Were between 6 and 18 years old.
(iii) Agreed to participate in the study in the presence of a witness, after having received an oral briefing and have written consent.

The sample size was calculated using a formula developed by Leslie Kish: *n* = *p*(1 – *p*)*z*^2^/*d*^2^ (*n* = required sample size, *z* = level of confidence, *p* = expected prevalence in proportion of one, *d* = precision in proportion of one). To obtain significant results for all parasites to be screened, we used the parasite with the lowest expected prevalence in our study region for p. For the study area, the prevalence of intestinal Protozoa was expected to be lowest (about 20%) [19]. We used the standard level of confidence of 95% (1.95). For *p*, we used the standard value of 0.05. Thus, a minimum sample size of 246 was calculated. In total, 307 children were participated in the initial screening process. After accounting for dropouts and incomplete data we obtained a sample size of 256.

Participation was voluntary and participants could leave the study at any stage without giving reasons.

## Data collection

### Questionnaire

We used a standardized questionnaire to record age, sex, height, and weight. The questionnaire included questions on nutritional status, living conditions, water- and washing patterns, education, and hygiene conditions. It was recorded, whether, and if so, when, the study participant had ever received anthelminthic treatment. The study participants answered the questions with the help of the local study team, who read the questions to the children if needed.

### Parasitological screening for Schistosomes and STH

A single stool and urine samples were collected in two separate clean containers from every study participant. On site, two Kato Katz thick smears were prepared from different sites of each stool sample, using a template of 41.7 mg (Vestergaard Frandsen, Lausanne, Switzerland), following the standard protocol [20]. The Kato Katz smears were examined for the presence of *S. mansoni* and STH eggs (*A. lumbricoides* and *T. trichiura* and Hookworms) by two trained laboratory technicians. For quality assurance, a random sample of 10% of the negative and positive Kato Katz thick smears was re-examined.

Aliquots of the stool samples were also conserved in formalin and in ethanol. The formalin-fixed samples were then concentrated with the SAF-method using an FPC-kit according to the manufacturer’s instructions. Two slides were prepared from each sample and covered with a coverslip (size: 22 × 32). The slides were then examined under the light microscope systematically by two trained laboratory technicians each using a 10 × and a 40 × objective.

### Parasitological screening for *S. haematobium*

All collected urine samples were examined for the presence of erythrocytes, protein and leucocytes using a urine dipstick test. Urine samples that were positive for hematuria underwent a urine filtration technique with Nuclepore^®^ membrane filters (Whatman International Limited, Maidstone, England), according to WHO standards for the presence of *S. haematobium* eggs [21].

### Circulating cathodic antigen test

In addition, all urine samples were tested with the *Schistosoma* Circulating Cathodic Antigens (CCA) Urine Cassette Assay (manufactured by Rapid Medical Diagnostics, Pretoria, South Africa) [22, 23]. Preparation and examination of urine samples for the CCA cassette were performed according to the manufacturer’s instructions. The entire laboratory technician team participating in reading the CCA results were blinded for the Kato Katz parasitological results of the study participants.

### Ultrasonography and clinical examination

Two medical doctors with extensive experience in clinical work and ultrasonography first conducted a physical examination and recorded any abnormalities. The study participants were then examined with a portable ultrasound machine in a supine position. The examining physicians were blinded to the participant’s *S. mansoni* and *haematobium* status.

The liver image pattern was obtained through a subcostal, transhepatic view, a substernal transverse view and sagittal scans. Spleen size, portal vein diameter and occurrences of ascites and other abnormalities were also recorded.

We used the simplified Niamey protocol as proposed by the German Society for Tropical Medicine and International Health to classify morbidity due to long-term Schistosome exposure. Liver image patterns type A + B were classified as non-periportal-fibrosis [24, 25].

### Data analysis

All data were double entered using Excel and the final data set stored in a MySQL database. The data were checked for consistency and any errors were cleaned. The statistical analysis was performed using IBM SPSS Statistics version 24 (SPSS Inc., Chicago, USA).

The data were analyzed using frequency tables, cross-tabulations and prevalence calculations. To compare the mean intensity of infection by sex, age, and group category, we used the* t* test and results were regarded as significant if *p* < 0.05. To obtain the number of eggs per gram of feces we multiplied the average number of eggs in each slide with 24 [25]. According to the WHO progress report, infection intensity with *S. mansoni* of 1–99 eggs per gram (epg) were classified as light, 100–399 epg as moderate and ≥ 400 as heavy.

To compare the appearance of periportal fibrosis (PPF) in either group, we used the chi-square test. Results were considered significant if *p* < 0.05. Liver image patterns (LIP) types A + B were considered as normal, C + D as mild, and E + F as severe patterns for periportal fibrosis [25].

### Treatment

After the examination and sample collection, all children received a single dose of Praziquantel, 40 mg/kg according to WHO recommendations [26] In addition to Praziquantel for the treatment of schistosomiasis, Albendazole 400 mg as a single dose was administered for the treatment of STH. To reduce the adverse effects of Praziquantel, a meal was provided for each study participant. After swallowing the drugs, participants were asked to remain on the field data collection point for 2 h, so that the research team could monitor and manage any adverse effects.

## Results

### Characteristics of study participants

#### Demographic information

144 orphans (70 (48.6%) males, 68 (47.2%) females, 6 (4.2%) unknown) and 112 street children (109 (97.3%) male, 3 (2.7%) unknown) took part in the study. The median age for orphans was 9 ± 3 years and for street children 13 ± 2 years.

**Fig. 1:**
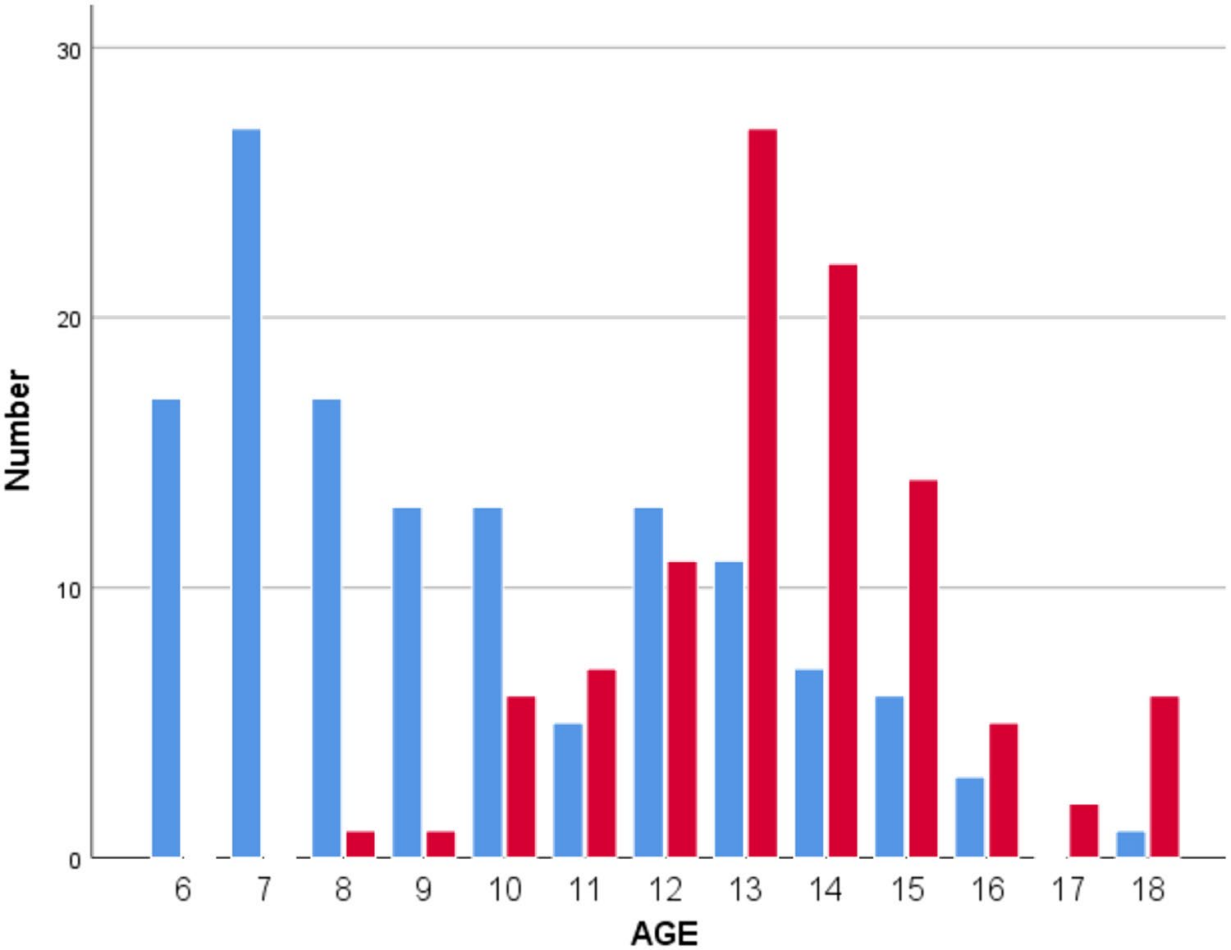
Age distribution of street children (red) and orphans (blue)

The median body mass index (BMI) in orphans was 16 ± 2.8 and in street children 17.15 ± 2. A lower BMI after regression for age and sex was significantly associated with infection status determined through the CCA test (*p* = 0.029) (Fig. 1).

#### Questionnaire

26.8% (66) claimed that they had received some form of anthelmintic treatment (22.8% of street children, 29.7% of orphans). 95.8% (138) of orphans stated that they attended school regularly. Only 12.7% (13) of the street children attended school regularly. While 100% of the orphans slept in orphanages, 14.9% (15) of the street children stayed with family overnight while the remaining children had no fixed place to stay overnight but slept in varying locations. All study participants expressed that they received drinking water from safe sources, namely piped water or wells. Only 1.4% (2) of the orphans stated in the questionnaire, that they used the lake to wash, whereas 97.1% (99) of the street children named the lake as at least one of their options for washing. 83.3% (80) of the street children do not regularly get food. Orphans solely used flush toilets or latrines. Of the street children 81.4% (91) stated in the questionnaire that they used “bushes” or “other” in addition to flush toilets or latrines.

### Ultrasonography

In only four of the participants (1.7%), the liver was palpable and in 100% of these cases the consistence was firm. One participant (0.4%) reported tenderness upon palpation. In four children (1.7%), the spleen was palpable and in one of those cases tenderness was reported.

Overall, we observed normal LIPs A + B (98.1%), mild PPF patterns C + D (1.9%) and no severe PPF patterns E + F. 118 (98.4%) of orphans and 87 (97.8%) of street children showed patterns A + B. 2 (1.6%) of orphans and 2 (2.2%) of street children had patterns C + D.

No significant correlation between LIP and sex, age or group category could be observed.

### Prevalence and intensity of infections with *S. mansoni* and *haematobium*

The prevalence for *S. mansoni* determined by POC-CCA-test was 65.9% (91) for orphans and 94.5% (103) for street children. After regressing for sex and group category, a higher age was significantly associated with infection status (*p* = 0.014). No significant difference between males and females could be observed with the limitation that there were no female street children in the study group.

Of the orphans, 19.2% tested positive for *S. mansoni in* Kato Katz (light = 64.6%, moderate = 17.7%, heavy = 17.7%). Of the street children, 77.1% showed positive test-results in Kato-Katz (light = 29.8%, moderate = 27.1%, heavy = 43.1%). After regression for sex and age, street children showed significantly higher infections compared with orphans (*p* = 0.003).

Having received anthelminthic treatment previously was negatively correlated with a positive test result in the CCA test (*p* ≤ 0.001). No significant correlation between STH infection rates and anthelmintic treatment was observed.

None of the children tested positive for *S. haematobium*.

### Prevalence of infections with STH and protozoa

Table 1 shows the infection rates of STH and protozoa as observed under the light microscope after concentration with the SAF-method. No significant correlation with sex, age, or group category could be observed. Overall, being a street child was significantly associated with being more likely infected with STH (*p* = 0.013).

**Table 1:** Number and percentage of orphans and street children who tested positive for STH and Protozoa

	Orphans (*n*^a^ = 61)	Street children (*n*^a^ = 74)
STH		
Hookworms	0% (0)	8.0% (9)
* T. trichiura*	0.7% (1)	1.8% (2)
* A. lumbricoides*	0% (0)	0% (0)
Protozoa		
* G. intestinalis*	3.4% (5)	5.4% (6)
* E. histolytica/dispar*	9.6% (14)	22.3% (25)

^a^Total number of valid stool samples obtained from orphans or street children

Microscopy showed positive results for *G. intestinalis* in 3.4% (5) and *E*. *histolytica/dispar* in 9.6% (14) of orphans. 5.4% (6) of the street children were positive for *G. intestinalis* and 22.3% (25) for *E. histolytica/dispar*.

### Prevalence of infections with STH

We found no infections with *A. lumbricoides.* Only one orphan (0.7%) and three street children (0.9%) were infected with *T.trichiura*. Nine (8.1%) street children and none of the orphans were infected with hookworms.

## Discussion

It is well established that many regions in sub-Saharan Africa suffer from high infection rates with Schistosomes [2, 15, 19]. Although there is a large amount of prevalence data across different populations, little information can be found on the prevalence in marginalized groups like street children and orphans.

A 2016 study in rural Tanzania, including study participants between the ages of 1 and 95 years, showed comparable results for infection rates with Schistosomes (Kato Katz 68.9%, POC-CCA 94.5%) to the rates we observed in street children. The infection rates for orphans in our study were lower than in the above study. This means that street children are exposed to a similar risk for infections as the general population in rural Tanzania, located on the shores of Lake Victoria. The study on Ijinga Island furthermore showed a high prevalence of related morbidities. PPF was associated with age; therefore, our study participants will likely experience a similar rise in morbidities with increasing age.

Apart from moral obligations to ameliorate the current situation for the children, there are also practical reasons. As with most public health issues, the problem of parasitic infections cannot be solved if certain groups of the population are exempt from interventions. Especially the lack of access to adequate sanitation like latrines for street children is concerning. Excretions of infected individuals that are not disposed of adequately help maintain the Schistosome lifecycle. The issue therefore not only presents a hazard for the health of street children but also for the rest of the population.

Chronic infections with Schistosomes in childhood can limit physical and cognitive development, which can reduce success at school and in the workplace. It is necessary to intervene during the critical stages of child development to avoid irreversible impairments. Especially for orphans and street children who are already disadvantaged in their prospects, including them in regular MDA programs could have an outsized impact.

In this study, we observed a high prevalence of infections with *S. mansoni*. The observed infection rates are significantly higher than in other studies conducted in Tanzania and neighbouring countries in school children. Our data indicate that there are differences in morbidity between orphans and street children. We observed a significantly higher rate of infections with *S. mansoni* in street children. Most street children compared with only a few orphans indicated in the questionnaire that they use the lakewater to wash, which could explain the results.

We did not find similar results concerning infection rates with protozoa. This suggests that both groups may live under comparable hygienic standards. Furthermore, all street children and orphans had access to safe drinking water. It is to note that mass drug administration programs usually treat helminth infections but not infections with protozoa. Therefore, better accessibility to MDA programs for orphans compared with street children would not influence the prevalence of infections with protozoa.

Surprisingly, we observed a low prevalence of STH. Given that the children spend a lot of their time outside and often walk barefoot we expected higher infection rates.

In the ultrasound, we found no severe LIPs of PPF. This can be expected as after the initial infection it takes on average 5–15 years for advanced fibrosis to manifest [27]. However some children already showed mild patterns of PPF. Given the high prevalence of Schistosomes in our study group with, in some cases, very high worm loads it can be expected that many will develop PPF, should they not benefit from regular interventions in the future.

There are some limitations to our study. We divided our study group into street children and orphans. However, it is difficult or even impossible to draw a clear line between both groups. Many of the street children have spent at least some time in an orphanage. Some street children are orphaned, some have run away from their families and others spend the day on the streets but return to their families at night. Furthermore, in our study, we only included male street children. The lack of female street children seems to be a general phenomenon. It might be due to females more frequently being forced into domestic or sex work and therefore disappearing from the streets, or that females run away from home less [18]. However, this characteristic complicates finding significant differences in the results. It is also to note that street children were on average four years older than the orphans. This could also influence the results, for example, through differences in risk behaviour. Furthermore, for each question, the children only had 2–6 possible answers to choose from. The data may, therefore, not portray a nuanced picture of the living circumstances of our study group. Due to financial constraints, we did not use polymerase chain reaction to differentiate between pathogenic and apathogenic types of Entamoeba. Thus, infections with protozoa were only used to determine the hygiene levels of the study participants. However, we cannot say whether any morbidity observed in this study was caused by protozoa infections. Furthermore, contrary to infection rates with Schistosomes, we did not see a significant negative correlation between STH infection rates and previous anthelmintic treatment. However, the questionnaire did not ask for specifications of which treatment they had received, as children will usually not know or remember the names of the medication. Therefore, we do not know whether the treatment included medication for both Schistosomes and STH.

Overall, we found that the general hygienic circumstances and access to healthcare of both groups could be substantially improved. As a first step, regular MDA campaigns that specifically target neglected groups in a population should be implemented. This could be achieved by including orphanages in MDA programs aimed at school children and building an infrastructure to offer treatment to street children at regular intervals. Particularly to reach street children, it is important to build trust in these intervention programs. This can be achieved by collaborating with NGOs like Tanzania Rural Health Movement that have experience working with these children and incentivising participation by offering free food and medical care. These public health measures would ameliorate the situation of the individuals treated and play a part in interrupting infection cycles. Furthermore, sanitation could be improved by building public latrines and facilities to wash with safe water. These public health measures are economical and easy to implement. By interrupting infection cycles the interventions could furthermore have an outsized positive impact on the health of a population.

## Conclusion

A high percentage of orphans and street children in Mwanza city is infected with one or more parasites. A significantly higher rate of infections with *S. mansoni* in street children compared with orphans could be observed. Infections with protozoa, which were used as a marker for hygiene, were on a comparable level for both groups. In ultrasonography, we observed no signs of severe PPF and only a few mild PPF patterns. Most street children use the lake to wash and often do not have access to adequate sanitation. However, everyone in the study group indicated having access to safe drinking water. Overall, we found the general hygienic conditions for both groups to be inadequate. With the help of simple public health measures, the overall situation could likely be considerably improved.

## Data Availability

The datasets used and/or analysed during the current study are available from the corresponding author on reasonable request.

## Abbreviations

*A. lumbricoides*: *Ascaris lumbricoides*
BMI: Body mass index
CCA: Circulating Cathodic Antigens
*E. dispar*: *Entamoeba dispar*
*E. histolytica*: *Entamoeba histolytica*
Epg: Eggs per gram
*G. intestinalis*: *Giardia intestinalis*
LIP: Liver image patterns
MDA: Mass drug administration
NTD: Neglected tropical disease
PPF: Periportal fibrosis
SAC: School-aged children
*S. haematobium*: *Schistosoma haematobium*
*S. mansoni*: *Schistosoma mansoni*
STH: Soil-transmitted helminths
*T. trichiura*: *Trichuris trichiura*
WHO: World Health Organization
